# The Influence of the Local Neighbourhood Environment on Walking Levels during the Walking for Wellbeing in the West Pedometer-Based Community Intervention

**DOI:** 10.1155/2012/974786

**Published:** 2012-07-29

**Authors:** L. B. Robertson, C. Ward Thompson, P. Aspinall, C. Millington, C. McAdam, N. Mutrie

**Affiliations:** ^1^OPENspace Research Centre, Edinburgh School of Architecture and Landscape Architecture (ESALA), University of Edinburgh, Lauriston Place, Edinburgh EH3 9DF, UK; ^2^Department of Sport, Culture and the Arts, University of Strathclyde, 76 Southbrae Drive, Glasgow G13 1PP, UK

## Abstract

We investigated the relationship between walking levels and the local neighbourhood physical environment during the Walking for Wellbeing in the West (WWW) randomised pedometer-based community intervention. Walking activity was recorded as step counts at baseline (*n* = 76), and at 3 months (*n* = 57), 6 months (*n* = 54), and 12 months (*n* = 45) post-intervention. Objective physical environment data were obtained from GIS datasets and street surveys conducted using the SWAT audit tool. Sixty-nine environment variables were reduced to eight environment factors using principal axis factoring, and the relationship between environment factors and (i) step counts, and (ii) the change in step counts relative to baseline, was examined using hierarchical multiple linear regression, controlling for age, gender, income, and deprivation. Five environment factors were significant predictors of step counts, but none were significant predictors of the change in step counts relative to baseline. None of the demographic variables included in the analysis were significant predictors at any stage of the study. Total variance explained by the environment ranged from 6% (*P* < 0.05) to 34% (*P* < 0.01), with lowest levels during the initial stages of the study. The physical environment appears to have influenced walking levels during the WWW intervention, and to have contributed to the maintenance of walking levels post-intervention.

## 1. Introduction

Physical activity is crucial for our health and well being, providing physiological and mental health benefits and helping to prevent chronic illnesses such as diabetes, cancer, and heart disease, which are the leading cause of death in most industrialised nations and many developing countries [1–3]. Walking is the most common form of physical activity among adults in many populations [4] and has been suggested as the mode of physical activity which is most likely to appeal to the least active of the population [5, 6]. Promotion of moderate physical activities such as walking is associated with longer-term changes in behaviour [7], and as such walking is increasingly highlighted in national and international physical activity health promotion strategies (e.g., [5, 8]). 

A number of individual, social, cultural, and economic factors affect physical activity levels, and the environment also plays a role [9]. Although causality between the physical environment and physical activity levels has yet to be established, there is now substantial evidence based on environment and physical activity and walking levels which indicates that the environment is an important contributory factor [9–13]. Policy and interventions to increase walking, therefore, need to target both people and places [14, 15], and it has been suggested that modifying the environment has the potential for much longer-lasting effects than individual level interventions, as environmental changes are assimilated into structures, systems, policies, and sociocultural norms [16], and thereby penetrate more widely and deeply into the complex socioecological system in which we live. 

The potential to create physical environments that support increased levels of activity is now being embraced, with many countries producing guidance on the creation and promotion of built and natural environments that encourage and support physical activity (e.g., [17, 18]). Environmental characteristics which have been identified to be positively associated with walking and/or physical activity in adults include aesthetics [19, 20]; safety from traffic [11]; residential density, land use mix, and street connectivity [21–23]; well-maintained footpaths [11, 24, 25] and street lighting [23]; the presence of facilities that function as destinations, for example, shops [11, 23–25]; access to facilities for physical activity for example, parks, and recreation centres [11, 12, 20, 24]; accessible, safe green spaces [26–29]. 

This paper reports on findings in the context of a walking intervention study and for a European city, for which there is a relatively limited evidence base. The study was carried out in Glasgow, UK, as part of the Scottish Physical Activity Research Collaboration (SPARColl) “Walking for Well-being in the West” (WWW) pedometer-based community walking intervention, conducted between August 2006 and October 2010 [30, 31]. The purpose of the current study was to investigate if characteristics of the environment around WWW particpants' homes was related to their walking levels, and to investigate if an environment seemingly more supportive of walking was associated with a change to, and maintenance of, higher levels of walking post-intervention. Identifying the relative importance of the environment compared to individual, social, or economic factors was beyond the scope of the study, but demographic variables known to have a bearing on walking levels were controlled for in the analysis [32]. 

## 2. Context: Walking for Wellbeing ****in the West (WWW)

The WWW study was designed to assess whether a pedometer-based walking programme in combination with physical activity consultations would increase walking over a 12 week (3 month) period, and whether any increases gained could be sustained at 12 months [30]. The study sample *(*n* = 79)* was drawn from men and women aged 18–65 years who were living in the west of Glasgow, Scotland, and who were not achieving the recommendation of at least 30 minutes of moderate-intensity physical activity on at least five days of the week [33]. Initially, the aim was to recruit participants from the lowest socioeconomic groups who lived within a 1.5 km radius of the University of Strathclyde Jordanhill campus, since areas of high deprivation are located in close proximity to this campus. However, due to recruitment of insufficient numbers, less deprived areas were also included in the study. The WWW intervention was delivered in two forms; a maximal and a minimal intervention. The maximal intervention consisted of a pedometer and a 12-week-individualised walking programme with graduated step count goals and additional behavioural and cognitive support via a series of physical activity consultations. As part of these physical activity consultations, participants were given a map of the local area with their home address highlighted. If participants found it helpful, this map was used to facilitate discussion of potential places or routes to walk in their local neighbourhood. Following a waiting-list control condition, the minimal intervention consisted only of the pedometer and walking programme. For the purpose of the study, participants were randomised into two groups: Group 1—immediate (maximal) intervention; Group 2—waiting list control (minimal intervention). Physical activity levels were assessed objectively, using pedometer step counts, and subjectively, using the International Physical Activity Questionnaire (IPAQ) [34]. Monitoring was carried out at baseline, and at 3 months, 6 months and 12 months post-intervention. Full details of the WWW study and design rationale can be found in Fitzsimons et al. [30]. The main findings to date are (i) the pedometer-based walking programme combined with a physical activity consultation was effective at promoting walking over 3 months [35]; (ii) anthropometric and metabolic measurements made during this time period showed that health outcomes remained stable [35, 36]; (iii) the minimal intervention was also successful at increasing step counts [31]; (iv) both groups maintained the increased step counts to 12 months, and both interventions were deemed cost effective [37]. Subjective physical environment data in the form of participants' perceptions of the local physical environment were obtained using the Neighbourhood Quality of Life Survey (NQLS, no date), IPAQ, and focus group discussions. Participants generally thought that characteristics of the built environment and safety in their neighbourhoods were important to support and enable active behaviour intentions and sustain longer term increases in activity and also felt that their neighbourhoods were supportive of walking [38]. 

## 3. Methods

### 3.1. Study Area and Population Sample

**Figure 1:**
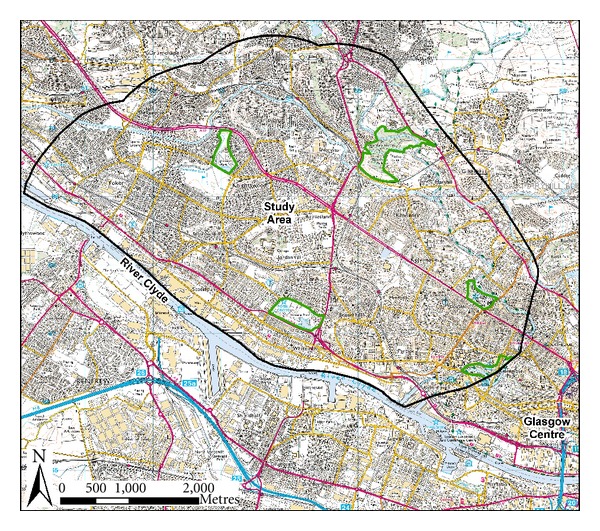
Location of the study area. Major green spaces within the study area are highlighted with a green border (Knightswood Park, Dawsholm Park, Victoria Park, Glasgow Botanics, and Kelvingrove Park). ©  Crown Copyright/database right 2008. An Ordnance Survey/EDINA supplied service.

**Figure 2:**
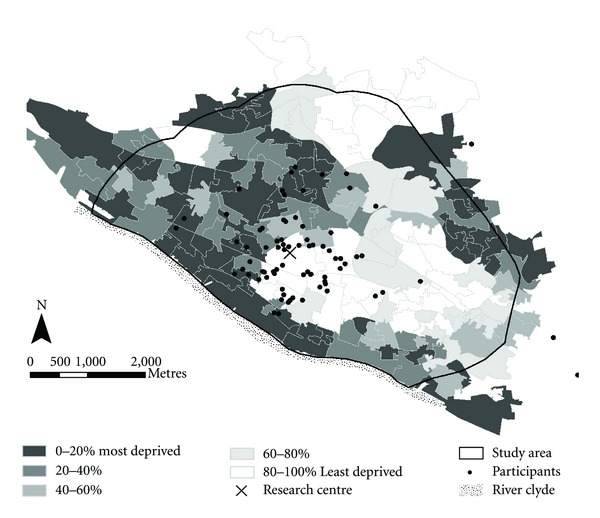
Participant locations and Scottish Index of Multiple Deprivation (SIMD) zones.

The WWW study site encompassed an area of approximately 25 km^2^ north of the River Clyde in Glasgow, Scotland (Figure 1). The land use of this area is predominantly residential, with some commercial destinations and industries bordering the river, and there are four main parks and a botanical garden. The site covers some of the most and least deprived areas within Scotland, based on the Scottish Index of Multiple Deprivation (SIMD) [39]. The location of participants and the SIMD zones for the study area are shown in Figure 2. At the time of participant recruitment (2006), the population density of the area was c. 3300 persons per km^2^. As three individuals lived outside the main study area, they were excluded from the environmental analysis reported here, giving a total adjusted sample of *n* = 76 at baseline, of which only 16 individuals were male. At baseline, the age of participants ranged from 27–66 years, with a mean age of 51 years (SD = 9). At 12 months post-intervention 45 participants returned (59%), and 13 of these were men. The mean age was 53 years (SD = 7.5, range 28–66 years). A significant challenge with a longitudinal study of this nature is to maintain participant numbers throughout the intervention and minimise drop out. Participants were lost from the study for a number of reasons including: being noncontactable; injury; personal reasons; lack of time; dissatisfaction with the pedometer [31].

### 3.2. Step Counts

Step counts were recorded using sealed Omron HJ-109-E pedometers (Omron Healthcare UK, Ltd.). The Omron pedometer includes a cover to prevent accidental resetting and has a 7-day memory, therefore, avoiding the need for participants to record their daily steps which can act as an incentive. Also, as the pedometers are sealed, no feedback is available whilst in use during monitoring. At baseline, participants were instructed to continue their normal activity levels and were asked to wear their pedometer continuously, except when sleeping, showering, or undertaking structured exercise. Any significant changes in step counts recorded should, therefore, largely reflect changes in walking behaviour [35]. Monitoring was conducted over a 1-week period on four occasions: baseline (*n* = 76), and at 3 months (*n* = 57), 6 months (*n* = 54), and 12 months (*n* = 45) post-intervention. In this controlled trial, the maximal intervention was successful in significantly increasing daily step-counts over 3 months by 3175 steps compared to a nonsignificant increase of 154 steps amongst those who were on a waiting list [35]. At 12 months, there was no significant difference between the walking levels of participants who received the maximal or minimal intervention, with both leading to an average increase of 1509 steps/day [31].

### 3.3. Environment Data

#### 3.3.1. SWAT Audit

The Scottish Walkability Assessment Tool (SWAT) [40], developed drawing on the work of Pikora et al. [41], was used to objectively record features of the physical environment which could be related to walking. The total audit area was defined by applying the audit tool to an area of 1600 m radius around each participant's home [42], a distance that could be accessed within approximately 30 minutes total walk time. Overlap of the 1600 m zones resulted in a continuous survey covering approximately 25 km^2^, which constitutes the current study area (Figure 1). Locations within the 1600 m zones south of the river were excluded from the study as the river provides a natural barrier to walking. Following the protocol developed by Pikora et al. [25], streets were divided into segments, defined as a length of street between two consecutive junctions. A total of 2030 street segments were audited during the summer of 2007, by three pairs of trained auditors. SWAT was designed to be administered from one side of the street (side 1), to describe separately the opposite side of the street (side 2). Further details of SWAT and the results of audit reliability tests can be found in Millington et al. [40]. Only audit items that were found to be reliably recorded were included in the current analysis, a total of 81 of 112 audit items, a number of which were combined to give meaningful environment characteristics/variables for the analysis (Table 1). Variables that were found to be unreliable were generally those which are subjective in nature and/or time dependent, for example, perceptions of safety and aesthetics. Methods of reducing the audit data to the initial set of variables used in the factor analysis (*n* = 56) are described in Section 3.3.3 below.

#### 3.3.2. GIS

A total of 13 environmental variables were derived from local and national GIS datasets and digital Ordnance Survey maps (Multipmap data from Digimap). Variables included measures of land use, residential density, street connectivity, and road accidents (Table 1). Land use data were obtained from the Macaulay Land Use Research Institute (now James Hutton Institute); road accident data (April 2004–March 2007) were supplied by the Strathclyde Police; bus stop data were provided by the Medical Research Council (Glasgow). The Scottish Index of Multiple Deprivation (SIMD) rank was also obtained [39], and values used in the analysis were for the SIMD data zone in which the participant resided. SIMD is a composite variable, derived from seven domains scores: income, employment, health, education, access, housing, and crime. Attempts to obtain more detailed crime statistics for use towards developing a separate “crime rate” variable proved unsuccessful. The SIMD measure, therefore, is the only indication in the study of crime as a variable. Given the environmental information that contributes to the access domain in SIMD, it was not included in the factor analysis of environment variables. 

#### 3.3.3. Summarising Street Audit and GIS Data for Individual Neighbourhood Zones

A circular neighbourhood zone of 400 m radius was defined for each participant, centred on their home. Although the audit data were initially collected for a radius of 1.6 km around participants' homes, these zones revealed considerable overlap, and so a 400 m radius zone was chosen for the analysis to reflect that of Pikora et al. [25], on whose work the SWAT audit tool had also been drawn. The 400 m radius zone was also chosen in order to maximise potential variability between neighbourhoods as low variability had been noted for a number of variables when assessing the reliability of the audit data [40]. A larger buffer zone, indicative of 10–15 mins walking distance, would have resulted in much reduced variability in characteristics between the defined “local neighbourhood” for each participant, and, therefore, of limited capacity to explain variability in walking levels. 

For each 400 m zone, the audit (*n* = 81) and GIS (*n* = 13) data were summarised for each segment that lay within or intersected with the 400 m neighbourhood zone. Then, depending on the nature of the data collected, a number of summary methods were used to obtain a single value for each environment characteristic/variable for each neighbourhood zone, resulting in a total of 69 environment variables for the analysis (56 from the audit data, and 13 from the GIS data).

**(i) Presence/absence variables**

For simple presence/absence data, in the case of GIS data, the number of items present in the neighbourhood was summed, for example, total number of bus stops. In the case of the audit data, the proportion of segments displaying a specific variable characteristic was calculated, for example, the proportion of segments in the neighbourhood with traffic signals. Where an item was recorded individually for both sides of the street, the data were combined to give a proportion for presence/absence on either side of the street.

**(ii) Interval variables from the audit data**

Interval data were summarised using a weighted average (variables denoted with an asterisk in Table 1). Audit items were coded with 0 or 1 as the lowest interval and up to a maximum of 8 depending on the number of intervals present for example, for the Garden maintenance variable: “>75% of gardens well maintained” was coded as 3; “50–75% of gardens well maintained” was coded as 2, and “<50% of gardens well-maintained” was coded as 1. Thus, in the above example, higher values equate to a greater proportion of the neighbourhood area with well maintained gardens. In the case of the Path material type variable, paths made from man-made materials (asphalt, paving blocks, paving slabs, setts, hoggin, and gravel) were assigned lower codes (1 to 6, resp.,) and paths made from natural materials (mud/earth/unpaved, grass) were assigned higher codes (7 and 8, resp.). Where interval variables were recorded individually for both sides of the street the mean of the weighted average for each side was used.

**(iii) Other variables**

Land use mix index was calculated for each 400 m radius zone as described by Frank et al. [21]. This variable represents the evenness of the distribution of domestic, commercial, and green space land use. Possible values range from 0 to 1, with higher values representing more mixed-use neighbourhoods. Dwellings per hectare values (derived from land use data) are for the SIMD data zone in which the participant resided. It was not possible to calculate summary values for the 400 m neighbourhood zones for dwelling density as the data available conformed to different boundaries.

### 3.4. Statistical Analysis

#### 3.4.1. Data Screening and Reduction

Prior to analysis the data were checked for normality (Shapiro-Wilk and ±2 × SE skewness normality tests) and screened for outliers (values greater than 3 × IQR removed). Data were transformed where necessary and possible (square root, natural log), and eleven environment variables were removed from the analysis on the grounds of there being (i) no data, (ii) duplication, or (iii) very low variability. Coach stop, Pool, and Zebra crossing were removed as they did not occur in any of the 400 m neighbourhood zones (i.e., the proportion of segments with these features was 0% for all participants). On the grounds of duplication between audit and GIS data, the street audit Bus stop variable was removed (GIS data preferred as they required less manipulation prior to analysis). Variables with a very low or high prevalence were removed, defined as those variables with >80% of the values for each participant/neighbourhood zone being equal to either 0% or 100% (Golf course, Bike locker, Underpass, and Path continuity). Proportion variables with a maximum value for all neighbourhood zones ≤2.5% were also removed (Bike rack, Paths area (%), and Train station). A total of 58 variables remained for the factor analysis (46 street audit variables and 12 GIS dataset variables).

#### 3.4.2. Factor Analysis

Principal axis factoring (PAF) was used for data reduction as some variables could not be transformed to a near normal distribution, and this approach is considered to be most appropriate for data with severe departures from normality [43]. The analysis was run with an eigenvalue of 1 and varimax rotation (SPSS v.18). After the initial run, the *Wild nature views* variable was removed because it was entirely correlated with *Nature views* (*Nature views* preferred on the basis that it would encompass *Wild nature views*). Individual variable sampling adequacy was tested using the Kaiser Meyer Olkin (KMO) criterion, leading to the removal of further 15 variables from the analysis, all with a KMO value <0.45. These were *Land uses (no. of)*, *Government buildings (mean no. of)*, *Hedge height*, *Bridge overpass*, *Road narrowing*, *Derelict land*, *Sports track*, *Parking on street amount*, *Verge maintenance*, *Tactile paving*, *Cycle lane*, *Crossing with lights*, *Path obstructions*, *Water views*, and *Commercial views*. On rerunning the analysis on the remaining dataset of 42 variables, all variables passed an individual sampling criterion of 0.5 (considered to be appropriate for a dataset of this size), and the overall sampling adequacy for all variables was very good (KMO = 0.707). The determinant of the correlation matrix was within limits, and Bartlett's test of sphericity was highly significant (*P* < 0.001), indicating that the analysis was appropriate for the dataset. Variables with loadings <0.5 were removed from the analysis (*Urban views*, *Street lights*, *Driveway crossovers,* and *Transport stops*), as for a sample size of *n* = 76 only loadings greater than about 0.5 are statistically significant (and thus account for variance in the dataset). Rerunning the analysis on the remaining 38 variable dataset produced a 9 factor solution with meaningful groupings. Factor 9 was removed from the analysis on the basis of an eigenvalue <1 and it consisting of only one variable loading at less than <0.5 (*Recreation facilities-mean no. of*). The final ratio of participants to variables was 2:1, making the analysis on the low side of acceptable based on sample size. Communalities were high however (0.642–0.983; mean = 0.841, SD = 0.09), and for most factors a number of variables loaded strongly (>0.5), indicating a reasonably strong dataset for factor analysis [44]. Factor scores were saved as Anderson-Rubin scores and prior to the regression analyses were checked for normality and outliers (data were transformed and outliers removed as described above). 

#### 3.4.3. Multiple Linear Regression Analyses

A hierarchical blocked regression was used (SPSS v.18, “Enter” method), with demographic variables entered in block 1, and the 8 environment factors in block 2. Demographic variables included were age, gender, income (annual household), and SIMD rank. The analysis was carried out for (i) step counts at baseline at each of the 3 monitoring periods post-intervention (3 months, 6 months, and 12 months); (ii) the change in step counts relative to baseline for each monitoring period post-intervention. Final models were checked for multicollinearity (variance inflation factor < 10). 

## 4. Results

**Figure 3:**
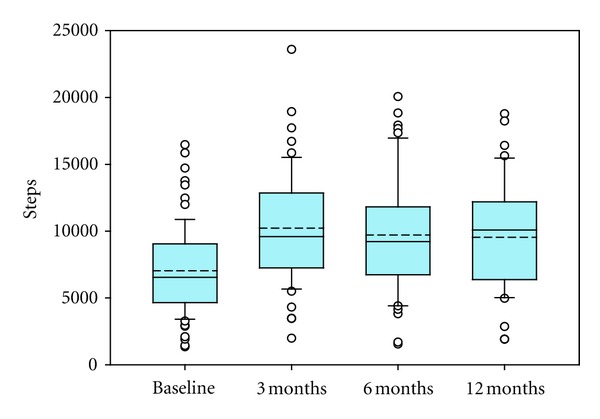
Step counts at each stage of the study. Whiskers are 10th and 90th percentiles and the dashed line is the mean.

Step counts at baseline and at each stage of the study post-intervention are shown in Figure 3. Step count followed an approximately normal distribution at 6 months and 12 months but were not normally distributed at baseline and 3 months. The median step counts at baseline was 6544 (IQR = 4396), which lies towards the upper end of the “low active” target group for participants [30, 45, 46]. After the intervention, median step counts increased by 46% (3 months) and then remained at approximately the same level above the baseline throughout the study (as noted above, see [31, 35]). Median step counts post-intervention were 9588 steps, 9221 steps, and 10085 steps, for 3 months, 6 months, and 12 months, respectively. The maximum level of activity for an individual over the length of the study was recorded at 3 months post-intervention (23589 steps), and the minimum was recorded at baseline (1346 steps). 

**Figure 4:**
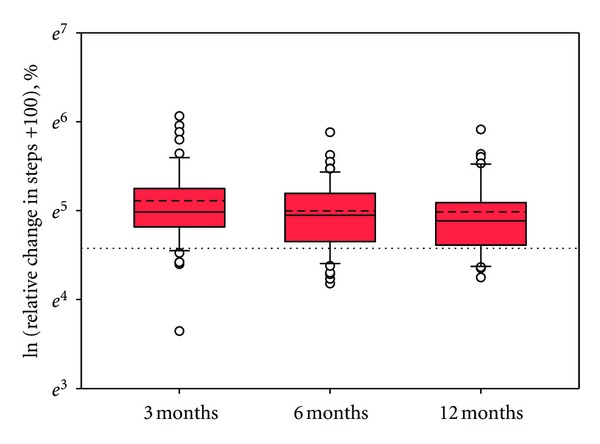
The relative change in steps from baseline at each stage of the study (NB: axis is natural log, with a constant of 100 added to all step counts). Whiskers are 10th and 90th percentiles and the dashed line is the mean. The dotted line at *e* ^4.6^ represents 0% change.

The relative change in step counts from baseline at each monitoring period is shown in Figure 4 (data followed an approximately log-normal distribution). The average relative change was largest at 3 months post-intervention (median = 46.1%), and smallest at 12 months post-intervention (median = 33.2%), as might be expected given the passing of time from the start of the study. The largest relative change observed in an individual was 330%, at 3 months. At each stage of monitoring, there were some participants whose activity levels fell below their baseline levels: the largest drop below baseline values was a decrease of 62%, for one participant at 3 months post-intervention. 

Table 2 shows the rotated factor matrix produced from the factor analysis. Only correlations >0.5 and those factors which were retained for the multiple regression analyses are shown. The total amount of variance explained by the 8 remaining factors was 80.7%. Factors were named, and the proportion of variance explained by each is, as follows: (1) *Green space and recreation facilities* (13.8%); (2) *Commercial and residential land use mix* (13.0%); (3) *Dangerous and busy roads (*12.6%); (4) *Pathway features other than safety* (12.3%); (5) *Pathway safety features* (9.7%); (6) *Roads and bus stops* (7.7%); (7) *Indoor fitness facilities and traffic calming features* (7.2%); (8) *Traffic signals and pedestrian signage* (4.2%). 

The results of the multiple linear regression analyses for step counts are shown Table 3. For the change in step counts relative to baseline, none of the demographic variables included or any of the environment factors were significant (*P < *0.05) predictors at any time period, and thus results are not reported. However, the* Indoor fitness facilities and traffic calming features* factor was borderline significant at 6 months, (*P* = 0.063, **β** =.27). For the step counts analyses, none of the demographic variables were significant predictors, but gender was borderline significant (*P = *0.058) at 3 months post-intervention. SIMD rank was also close to significance at 3 months (*P = *0.096), and income was close to significance at 12 months (*P = *0.097). At each stage of the study, one or more environment factors were significant predictors. The total amount of variance which could be explained by the environment factors varied over the length of the study, ranging from 6% (*P < *0.05) at baseline to a maximum of 34% at 6 months (*P = *0.001, dropping to 28% when adjusted for a population study). Different factors were found to be significant at each time period, with the exception of at baseline and at 3 months, when results were consistent. At baseline and at 3 months, the *Dangerous and busy roads *factor was the only significant predictor (*P < *0.05) and was inversely related to step counts. The total amount of variance accounted for by this factor at baseline and at 3 months was 6% and 8%, respectively (*P < *0.05). At 6 months, four of the environment factors were significant predictors and together these accounted for 34% of the variability in step counts (*P = *0.001). The *Commercial and residential land use mix* factor was the most important and was positively associated with step counts (*β* =  .40). The remaining three factors were of approximately equal importance, with the *Dangerous and busy roads *and *Traffic signals and pedestrian signage* factors inversely related to step counts (*β* = −.31 and −.30, resp.), and the *Indoor fitness facilities and traffic calming features* factor was positively related to step counts (*β* =.27). At 12 months, the *Green space and recreation facilities* factor was a significant predictor (*P < *0.05), and this was an inverse relationship (*β* = −.34). The *Commercial and residential land use* factor showed a borderline significant (*P = *0.05) association with step counts, and this was a positive association, as found at 6 months. Together, the two factors accounted for 19% of the variance in step counts (*P < *0.05). 

## 5. Discussion

### 5.1. Characteristics of the Environment Associated with Walking

Over the course of the study different aspects of the environment were found to be influencing walking levels at different times, but in all cases the direction of associations were consistent over time (Table 3). The five environment factors that were found to be significant predictors of step counts were *Dangerous and busy roads* (inversely related)*, Commercial and residential land use mix *(positively related), *Indoor fitness facilities and traffic calming features *(positively related)*, Traffic signals and pedestrian signage *(inversely related), and *Parks and recreation facilities* (inversely related). The pathway features factors (factors 4 and 5) and the *Roads and bus stops* factor (factor 6) were not significant predictors. 

The inverse association between step counts and the *Dangerous and busy roads *factor, which was a significant predictor at baseline, and at 3 months and 6 months post-intervention (all *P < *0.05), shows that walking levels were lower in neighbourhoods with a higher density of traffic, and this was despite higher levels of garden maintenance generally also being present in these neighbourhoods (Table 3). Previous studies have shown the impact of traffic density on walking to be mixed, due to its association with street network permeability and access to amenities. For example, Giles-Corti and Donovan [47] found a positive association between walking for transport and perceiving traffic to be present and heavy, but Cao et al. [48] found that perceiving traffic to be present and heavy seemed to discourage both transport and recreation walking, and Duncan et al. [11] found that physical activity was more likely where traffic is not perceived to be a problem. In addition to concerns for safety, poorer air quality and higher noise levels associated with heavy traffic may also discourage walking in neighbourhoods with a higher density of traffic and busy roads [49]. The inverse association found for the *Traffic signals and pedestrian signage *factor, which was a significant predictor of step counts at 6 months, probably also reflects an aversion to busy roads, as a greater incidence of both of these features can generally be associated with a higher density road network.

The positive relationship observed between step counts and the *Commercial and residential land use mix* factor, which was a significant predictor of step counts at 6 months (*P <* 0.05) and 12 months (*P = *0.05) post-intervention (Table 3), is in concordance with the observations of a positive association between walking and land use mix and a high density of shops/amenities consistently reported in the literature [9, 10, 13]. A positive relationship was also observed between step counts and the *Indoor fitness facilities and traffic calming features* factor, which was found to be a significant predictor at 6 months post-intervention (Table 3). Access to recreation and sports facilities have generally been found to be positively associated with increased physical activity [9, 10, 13], as would be expected, especially as these types of centres often provide additional facilities that can act as walking destinations, for example, cafes. Inverse associations have been noted by others, however, for example, Giles-Corti and Donovan [50] found that members of recreation and sports clubs were only half as likely to achieve recommended walking levels than those who were not club members. Traffic safety measures have been found to be positively associated with physical activity (e.g., [11]), as would be expected due to the decreased risk of road accidents and a more attractive environment for walking associated with lower driving speeds. Further, Morrison et al. [51] found the introduction of traffic calming measures to have a positive impact on physical activity levels of a Glasgow community, based on observations of pedestrian activity made before and after the changes were made. A corresponding significant improvement in physical health was also noted (measured using the SF-36 instrument). 

The inverse relationship between walking levels and the *Green space and recreation facilities* factor at 12 months post-intervention (*P* < 0.05) is somewhat contrary to what would be expected given the generally positive association found between physical activity and recreation facilities, discussed above, and the large number of studies which have shown a positive association between physical activity levels and accessible, safe green spaces (e.g., [26–29]). Inverse associations between walking and green space have also been found elsewhere, however [10, 13]. Safety concerns, poor quality green space, and low perceived accessibility are factors which could account for this pattern [52–55], and several studies suggest that any association between residential proximity to green space and health is more strongly associated with mental than with physical health (e.g., [56, 57]). Given that all four parks in the study area sit adjacent to some of the most deprived areas in Scotland (an SIMD rank in the lowest two quintiles (0–40%), Figures 1 and 2), and that the majority (71%) of study participants were female, it seems plausible that the inverse association observed here could at least in part reflect safety concerns, as safety has been found to be more important for woman's physical activity levels than men (e.g., [58]). The quality of the parks and green spaces was not audited as part of this study and it may be that nearby green space considered of poor quality is a deterrent to use for walking, as suggested by other research [53]. Alternatively, it may be that low perceived accessibility is a barrier to use, rather than poor quality, for example, in a Glasgow study Macintyre et al. (2008) found that a park of good quality may not be visited by people from deprived areas for this reason. Sugiyama et al. [53] suggest that distance is not the only factor in the association between walking levels and neighbourhood green space, especially if the purpose of the visit is recreational walking, and that quality (attractiveness) and size of the park may override distance in importance. This study also suggested that nearer local parks may be visited more often, but used in a less active way, perhaps for mental relief and relaxation rather than physical activity. Thus, the inverse association with walking activity levels observed here could be an artefact of the neighbourhood scale used in this study (400 m radius). Residential density (*Dwellings per hectare)* was also a component of the *Green space and recreation facilities* factor (Table 3) and is, therefore, also inversely related to walking levels. Again, this is contrary to what would be expected based on previous studies [10, 13]. It is possible that this finding may reflect a tendency for the highest residential densities to be found in the most deprived areas, which are generally associated with lower levels of physical activity/walking.

The lack of any significant association between step counts and the *Pathway features other than safety*, *Pathway safety features*, and the *Roads and bus stops* factors suggests that these aspects of the physical environment were not important factors influencing walking levels during this study. This probably reflects the overall quite low level of variability in these features across the study area [40], and that neighbourhoods are generally supportive of walking in terms of these features.

### 5.2. Relative Importance of the Environment over Time

The amount of variability in walking levels which could be accounted for by the environment factors varied over the course of the study, from a maximum of 34% at 6 months post-intervention, to a minimum of 6% at baseline (Table 3). The much larger total variance accounted for at 6 months compared to at baseline and 3 months (6% and 8%, resp.) suggests that the environment became a more important influence on walking levels as time passed, but it was not a major factor in the early stages of the study. This pattern is what might be expected given the context of this study, as at baseline walking levels were low (and thus exposure to the outdoor environment), and individual and social factors such as perception, motivation, self-efficacy and social support are known to be more important factors for behaviour change (e.g., [59–61], and therefore would be expected to account for more of the variability in walking activity during the initial stages of the study. Thus, these findings suggest that environmental factors are unlikely, on their own, to be influential in walking behaviour change but they may contribute to the maintenance of higher walking levels as time passes post-intervention. The lower amount of variance explained at 12 months post-intervention compared to at 6 months cannot be accounted for by a decline in walking levels/reduced exposure to the outdoor environment, as average step 26 counts were almost equal (Figure 3; 6 month mean = 9658 steps, SD = 4282; 12 month mean = 9677 steps, SD = 4001). This pattern would suggest that there was an increase in the relative importance of other factors which influence walking activity in the later stages of the study, such as motivation levels and changes in perception of the environment, or seasonal variations in walking activity related to changes in weather and day length may have caused this shift. The latter is not straightforward to analyse, however, due to a rolling recruitment programme with interventions starting on different dates over a 6 month period from August to December of 2006, and thus different seasonal effects for individuals at different stages in their personal programme of interventions. All we can conclude from the current analysis is that any identified influence of environment on walking levels is found despite any potential variability in effects of seasonality or weather. 

### 5.3. Change in Walking Levels Relative to Baseline

That no significant relationship was found between the environment factors and the relative change in step counts suggests that the local physical neighbourhood environment was not a factor influencing the change in physical activity levels in this study, although (on the basis of the discussion above), it appears to have contributed to maintenance of post-intervention walking levels over time (Table 3). As discussed above, given the importance of psychological and social factors for behaviour change, (e.g., [54, 60, 61]), this might be expected; however, there are a number of limitations relating to participant characteristics and the data collected during this study which might also account for this outcome. Firstly, due to problems with recruiting from the more deprived areas and the inclusion of more affluent areas in the study (Figure 2), it is possible that there were fewer environmental constraints on walking than if all participants had been from highly deprived areas. Because of this, it is possible that there may not have been enough particularly unsupportive neighbourhoods present in the study to be able to capture an environmental influence. This is supported by findings from the qualitative analysis, namely, that participants generally felt that their neighbourhoods were supportive of walking [38]. Secondly, it is possible that the change in step counts from baseline were too small overall for an environment effect to be adequately detected. As baseline walking levels of study participants were in general towards the upper bound of being considered “low-active,” this also seems plausible. Although some large relative increases in step counts were observed for certain individuals at each stage of the study (Figure 4), average (median) values were more modest, ranging from 33.2% at 12 months post-intervention to 46.1% at 3 months post-intervention. 

### 5.4. Contributions of This Study and Further Work

As far as we are aware this study is the first to have examined the influence of the physical environment on walking levels in the context of a walking intervention. It is one of only a few studies to provide information on walking-physical environment relationships for a European city, and whilst many studies have investigated associations between walking activity and characteristics the built environment, relatively few have employed factor analysis data reduction methods to help identify relationships with underlying, or composite, environmental variables [62, 63]. Statistical data reduction techniques are useful and preferable as they introduce analytical rigor to the analysis and thereby improve the reliability and validity of research findings [64]. The environmental factors produced from our analysis map to expectations based on the literature to some extent; however, due to the challenges in producing reliable, independent audit scores for perceived safety and aesthetics in the urban context of our study, the absence of safety and aesthetics variables is notable. It can be conjectured that residents' self-report perceptions of safety and aesthetics will vary from those of independent auditors in any case, as other studies have shown [9], and that residents' perceptions of safety, in particular, are likely to be more meaningful. 

The findings of this research also make a useful contribution to the knowledge base on walking activity and urban design and management. The results suggest that environmental factors contribute to the context in which healthy walking levels may be attained and maintained but that other individual and social factors may be the dominant influence, particularly in relation to interventions to increase walking, depending on time and circumstance. The evidence on the change in relative importance of the environment over time, after a pedometer-based intervention, suggests that certain aspects of the environment that are supportive of walking become increasingly important in the first year following such an intervention. The environmental factors that support walking, in the Glasgow context of this study, are a mix of different commercial and residential land uses, traffic calming measures, and the availability of indoor fitness facilities. Dangerous, busy roads and the need for traffic lights are inhibitors of walking, as are certain parks and recreation facilities; it seems likely that poor quality green space and/or low perceived accessibility or safety of parks and recreation facilities are the reason for the negative association found here, but more research is needed to confirm this. Overall, the evidence points to aspects of the Glasgow environment whose modification might be expected to make a difference to walkability and, therefore, to walking levels.

This study has also demonstrated that the SWAT street audit is a potentially useful tool for characterising the neighbourhood environment, but it appears that not all of the features included are relevant for assessing variations in walkability between the different parts of the Glasgow context. This may be because of insufficient variation in some aspects of the environment under study, so that attributes that are supportive of walking (or inhibit it) are present in almost all cases, and, therefore, their significance has not been detected. The presence of roadside pavements (sidewalks), for example, is almost universal in Glasgow. It is important, therefore, to recognise that environmental attributes that have not been identified as significant in relation to variations in walking levels across particular locations may nonetheless be a vital contributor to the necessary conditions for a walkable environment. Such attributes may not be sufficient to enhance or inhibit healthy walking levels in the absence of other interventions, such as social support, but they may be necessary for those interventions to have an effect [14]. To understand these factors better, SWAT should now be tested with other longitudinal studies, ideally in other UK and other European cities, where a diversity of environment will help to tease out the environmental attributes that are important for different contexts. For any further studies conducted in Glasgow, it may be effective to limit future audits to those elements which were found to be significant in association with walking. However, the full audit tool is likely to be useful in a different urban environment, where the environmental characteristics might vary much more widely, and different elements be shown to be significant for variations in walking levels. Also, as with any audit tool, SWAT will be most useful in combination with GIS data for environmental characteristics that cannot be captured effectively using the audit tool. To add to our understanding of the Glasgow context, further analysis of the physical environment data in combination with subjective walking data from IPAQ, in particular information on actual walking routes and walking purpose, may be able to provide a deeper insight into the relationship between environment and walking levels during the WWW study. 

## 6. Conclusions

This study has shown that certain characteristics of the physical environment of local Glasgow neighbourhoods appear to have influenced walking levels during a pedometer-based community intervention to increase walking, and that the relative influence of the environment varied over time. The environment was not an important factor influencing the change in walking levels; however, it appears to have contributed to the maintenance of post-intervention walking levels over time, for up to a year post-intervention. Factors such as land use mix, traffic levels, and traffic calming, and the quality and accessibility of recreational facilities and green space, have been identified as elements of the environment which contribute positively or negatively to walkability, and, therefore, are potential targets for better planning, design, and management. This study has also demonstrated that the SWAT street audit tool has good potential for characterising neighbourhood environments, and it should now be tested with other longitudinal studies, ideally in other UK and European cities.

## Figures and Tables

**Table 1:** Environment variables included in the analysis (*n* = 69), arranged according to theme and element after Pikora et al. [25]. GIS data variables (i.e. those not derived from the street audit data) are shown in italic (*n* = 13), and variables that were included in the factor analysis are shown in bold (*n* = 58). Variables which are weighted scores are indicated with an asterisk. Unless otherwise stated (e.g. %, number, mean), the values used in the analysis were the proportion of segments in the 400 m radius neighbourhood zone where the environmental feature in question was present.

Theme	Functional				Safety		Aesthetic		Destination		
Elements	Walking surface	Streets	Traffic	Permeability	Personal	Traffic	Streetscape	Views		Facilities	

Items	Path continuity	**Car lanes (mean no. of)**	**Traffic signals**	*Paths area (%)*	**Hedge height**	***Accidents injury (no. of)***	**Garden maintenance** ^ ∗^	**Hill views**	***Land use mix index*** ^ b^	**Park**	***Bus stops (no. of)***
	**Path obstructions**		**Driveway crossovers**	***Paths and roadside area (%)***	**Street lights**	***Accidents injury to pedestrians (no. of)***	**Hedge % coverage** ^ ∗^	**Nature views**	***Land uses (no. of)***	**Playground**	Bus stop
	**Bridge overpass**		**Road narrowing**	***Junctions:three legged (no. of)***	**Path well lit**	***Accidents noninjury (no. of)***	**Verge maintenance** ^ ∗^	**Wild nature views**	***Green space area (%)***	**Sports/playing field or tennis court**	Coach stop
	Underpass		**Speed humps**	**Steet closing but walking access through**	**Road names visible**	**Crossing with lights**	**Derelict land**	**Water views**	***Commercial area (%)***	**Sports track**	Train station
	**Path: none**		**Cycle lane**	**Cul de sac or permanent street closing** ^ a^	**Pedestrian signage**	Zebra crossing	**Dog fouling** ^ ∗^	**Urban views**	***Domestic area (%)***	Pool	**Transport stops (mean no. of)**
	**Path material type** ^ ∗^		Bike locker			**Median refuge**		**Commercial views**	***Dwellings per hectare***	Golf course	
	**Path material natural**		Bike rack			**Path distance from kerb** ^ ∗^			**Government buildings (mean no. of)**	**Fitness facility: indoor**	
						**Raised kerb**			**Parking provision** ^ ∗^	**Recreation facility: other** ^ c^	
						**Kerb extension**			**Parking on street amount** ^ ∗^	**Recreation facility (mean no. of)**	
						**Tactile paving**					

^a^At either end of the segment.

^b^After Frank et al. [21].

^c^Recreation facility other than an indoor fitness facility, park, playground, pool, golf course, sports/playing field, sports track or tennis court.

^∗^Variable is a weighted score.

**Table 2:** Rotated factor matrix for environment variables (*n* = 38), based on Principle Axis Factoring and a varimax rotation (with Kaiser normalisation). Only retained factors and loadings > 0.5 are shown. GIS variables (i.e. those not derived from street audit data) are shown in italic, and variables which are weighted averages are indicated with an asterisk.

Physical environment variable	Factor loadings							
	1	2	3	4	5	6	7	8
	Green space and recreation facilities	Commercial and residential land use mix	Dangerous and busy roads	Pathway features other than safety	Pathway safety features	Roads and bus stops	Indoor fitness facilities and traffic calming features	Traffic signals and pedestrian signage
Park	.924							
Recreation facility: other^a^	.899							
Playground	.652							
*Dwellings per hectare*	.631		.511					
*Paths and roadside area *(%)	.629							
Recreation facilities (mean no. of)	.608						.550	
Nature views	.532							
*Land use mix index* ^ b^		.924						
*Green space area *(%)		−.877						
Parking provision^∗^		.693						
*Commercial area *(%)		.644			−.501			
Hedge % coverage^∗^		−.606		−.514				
Cul de sac or perm. street closing^c^		.540						
Path: none		.531						
*Bus stops (no. of)*		−.511				.506		
*Accidents: injury (no. of)*			.900					
*Accidents: inj. to pedestrians (no. of)*			.869					
*Accidents: non-injury (no. of)*			.791					
Garden maintenance^∗^			.675					
*Junctions: three-legged (no. of)*	.504		.541					
Pedestrian signage			−.536					.510
Dog fouling^∗^			−.534					
Path material type^∗^				.865				
Path material natural				.860				
Sports/playing field or tennis crt.				.693				
Hill views				−.646				
Road names visible				.601			.528	
Street closing w. walking access thr.				.519				
Path well lit					.847			
Raised kerb					.797			
Path distance from kerb^∗^	.517				.591			
*Domestic area (%)*					.580			
Median refuge						.919		
Car lanes (mean no. of)						.798		
Fitness facility: indoor							.881	
Speed humps					.567		.643	
Kerb extension							.553	
Traffic signals								.710
**Percent of variance **	**13.8**	**13.0**	**12.6**	**12.3**	**9.7**	**7.7**	**7.2**	**4.4**
**Total variance explained = 80.7%**								

^a^Recreation facility other than an indoor fitness facility, park, playground, pool, golf course, sports/playing field, sports track, or tennis court.

^b^After Frank et al. [21].

^c^At either end of the street segment.

**Table 3:** Multiple linear regression analysis predicting step counts at baseline and at each monitoring period postintervention.

	Baseline^2^			3 months^2^			6 months			12 months		
	b	SE b	*β*	b	SE b	*β*	b	SE b	*β*	b	SE b	*β*
*Step * 1												
Age^1^												
Gender												
Income (annual household)												
SIMD rank												
*Step * 2												
Green space and recreation facilities (*Fac. 1*)^1^										−12667.3	5411.4	−.34^∗^
Commercial and residential land use mix (*Fac. 2*)							1896.3	582.7	.40^∗^	1259.5	622.4	.30^†^
Dangerous and busy roads (*Fac. 3*)^1^	−23.7	11.2	−.25^∗^	−28.0	13.7	−.28^∗^	−6376.0	2574.6	−.31^∗^			
Pathway features other than safety (*Fac. 4*)^1^												
Pathway safety features (*Fac. 5*)												
Roads and bus stops (*Fac. 6*)												
Indoor fitness fac. and traffic calming feat. (*Fac. 7*)^2^							5576.3	2512.4	.27^∗^			
Traffic signals and pedestrian signage (*Fac. 8*)							−1295.7	531.7	−.30^∗^			
Constant	44.3	18.0		55.1	22.1		−12709.5	6992.2		39003.28	12478.1	
*R* ^2^ Step 1 (adj. *R* ^2^)												
Δ*R* ^2^ Step 2 (adj. *R* ^2^)												
Model / Step 2 *R* ^2^(adj. *R* ^2^)	0.06 (0.05)^∗^			0.08 (0.06)^∗^			0.343 (0.283)^∗∗^			0.19 (0.15)^∗^		
Model *P*	0.038			0.046			0.001			0.018		

^1^Natural log transform; ^2^Square root transform

*<0.05. **<0.01.

^†^Borderline significant, *P* = 0.05.
